# Laparoscopic radical antegrade modular pancreatosplenectomy: preliminary experience with 10 cases

**DOI:** 10.1186/s12893-021-01090-w

**Published:** 2021-02-10

**Authors:** Ren-Chao Zhang, Xin-Jun Gan, Wei Song, Song-Tao Shi, Hui-Fang Yu, Yi-Ping Mou

**Affiliations:** 1 Department of Gastrointestinal & Pancreatic Surgery, Zhejiang Provincial People’s Hospital, Key Laboratory of Gastroenterology of Zhejiang Province, People’s Hospital of Hangzhou Medical College, 158 Shangtang Road, Hangzhou, 310014 Zhejiang China; 2 Department of Gastrointestinal Surgery, The First People’s Hospital of Jiashan, 1218 Tiyu Road, Jiashan, 314100 Zhejiang China

**Keywords:** Pancreatic cancer, Laparoscopy, Radical antegrade modular pancreatosplenectomy

## Abstract

**Background:**

The radical antegrade modular pancreatosplenectomy (RAMPS) which is a reasonable surgical approach for left-sided pancreatic cancer is emphasis on the complete resection of regional lymph nodes and tumor-free margin resection. Laparoscopic radical antegrade modular pancreatosplenectomy (LRAMPS) has been rarely performed, with only 49 cases indexed on PubMed. In this study, we present our experience of LRAMPS.

**Methods:**

From December 2018 to February 2020, 10 patients underwent LRAMPS for pancreatic cancer at our department. The data of the patient demographics, intraoperative variables, postoperative hospital stay, morbidity, mortality, pathologic findings and follow-up were collected.

**Results:**

LRAMPS was performed successfully in all the patients. The median operative time was 235 min (range 212–270 min), with an EBL of 120 ml (range 100–200 ml). Postoperative complications occurred in 5 (50.0%) patients. Three patients developed a grade B pancreatic fistula. There was no postoperative 30-day mortality and reoperation. The median postoperative hospital stay was 14 days (range 9–24 days).The median count of retrieved lymph nodes was 15 (range 13–21), and four patients (40%) had malignant-positive lymph nodes. All cases achieved a negative tangential margin and R0 resection. Median follow-up time was 11 months (range 3–14 m). Two patients developed disease recurrence (pancreatic bed recurrence and liver metastasis) 9 months, 10 months after surgery, respectively. Others survived without tumor recurrence or metastasis.

**Conclusions:**

LRAMPS is technically safe and feasible procedure in well-selected patients with pancreatic cancer in the distal pancreas. The oncologically outcomes need to be further validated based on additional large-volume studies.

## Background

The radical antegrade modular pancreatosplenectomy (RAMPS) for left-sided pancreatic cancer was initially described by Strasberg et al. [1] in 2003. This procedure was emphasis on the complete removal of regional lymph nodes and tumor-free margin resection [1]. RAMPS was expect to obtain high negative tangential margins rate and a favorable survival rate [1–4]. Laparoscopic pancreatic surgery has been gaining popularity in the last two decade due to recent technological developments in laparoscopic technique and instruments. However, Laparoscopic radical antegrade modular pancreatosplenectomy (LRAMPS) has been rarely performed, with only 49 cases indexed on PubMed [5–14].

There are still some concerns about the feasibility and safety of this technique. The best surgical procedure of LRAMPS is not yet established. The aim of this paper was to present our experience of 10 cases of LRAMPS.

## Methods

From December 2018 to February 2020, 10 patients underwent LRAMPS for pancreatic cancer at the our institute. The preoperative assessment included laboratory examination, computed tomographic (CT) scan, magnetic resonance imaging (MRI), endoscopic ultrasound (EUS) or fine-needle aspiration (FNA), and positron emission computed tomography.

The data studied were the patient demographics, intraoperative variables (operative time, estimated blood loss (EBL), conversion to open operation, blood transfusion requirement), postoperative hospital stay, morbidity, mortality (within 30 days from surgery), pathologic findings (tumor size, count of retrieved lymph nodes, margin status) and follow-up.

Pancreatic fistula (PF) was assessed according to the International Study Group on Pancreatic Fistula recommendations [15]. PF grade A was considered an asymptomatic biochemical leak and not counted as a complication, according to the modifications of the International Study Group definition of PF [16].

Patients were followed up via out-patient examination. The final follow-up was taken in February 2020. Recurrence or distant metastasis was diagnosed pathologically by surgical resection, biopsy, or cytology and/or radiological examination.

The Institutional Review Board of Zhejiang provincial people’s Hospital and The First people’s Hospital of Jiashan approved this study. The written informed consent was obtained from the patients before inclusion in the study.

### Operative technique

Patients were placed in supine position with the head slightly elevated. The surgeon and the second assistant who held the laparoscope stood on the right side of the patient and the first assistant stood on the left. One initial 10-mm trocar was placed for laparoscopy below the umbilicus. A 30° telescope was inserted to examine the peritoneal cavity to rule out metastatic disease. After general exploration, the other four trocars (one 12 mm, three 5 mm) were inserted into the left upper flank, left flank, right upper flank, and right flank quadrants; the five trocars were arranged in a V shape.

**Fig. 1:**
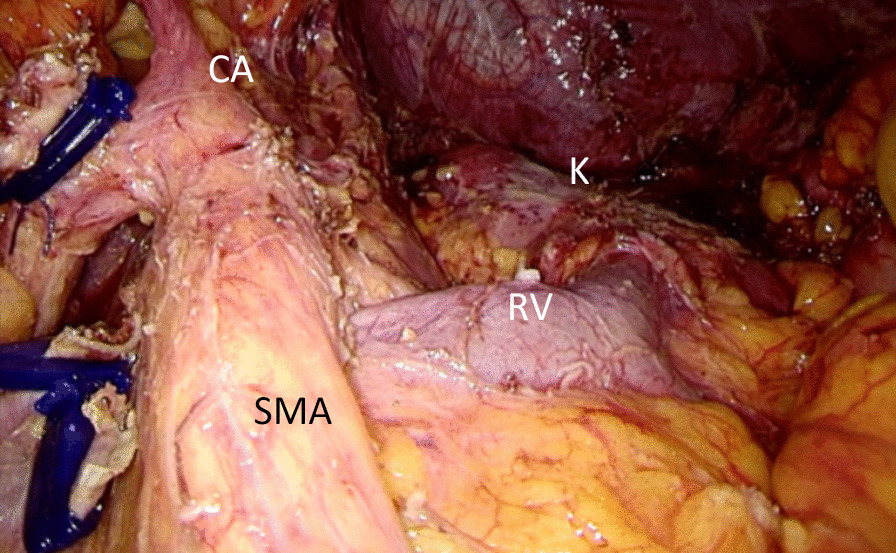
Final view after Laparoscopic radical antegrade modular pancreatosplenectomy. *CA* celiac artery; *K *kidney, *RV* renal vein, *SMA* Superior mesenteric artery

**Fig. 2:**
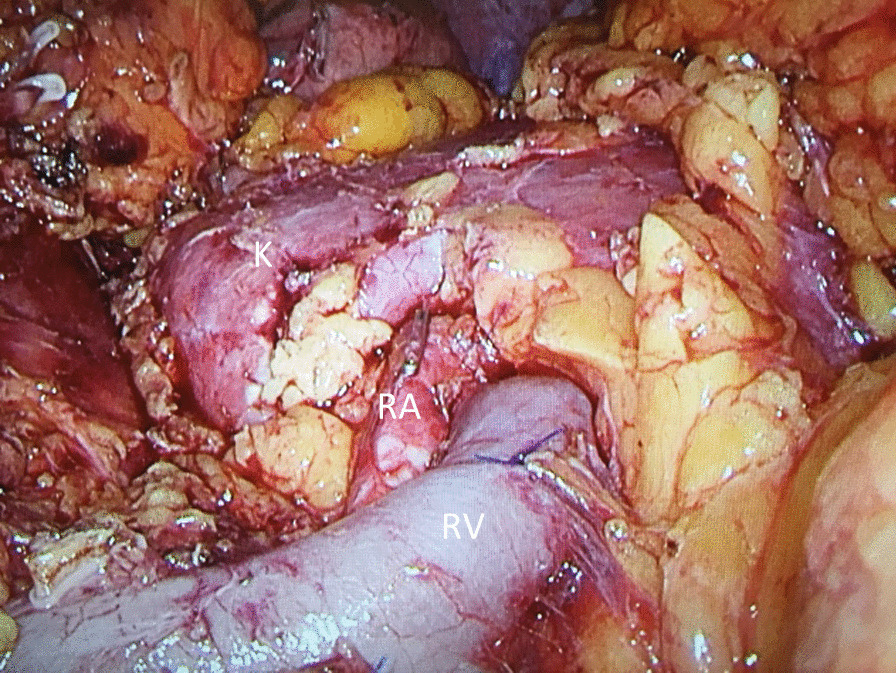
Final view after Laparoscopic radical antegrade modular pancreatosplenectomy. *K *kidney, RA renal artery, *RV *renal vein

The gastrocolonic ligament was divided for entrance to the lesser sac with harmonic scalpel (Harmonic Ace scalpel, Ethicon Endo-Surgery, Inc, Cincinnati, OH, United States). The mobilization of the pancreas began at the inferior border to visualize the superior mesenteric vein the splenomesenteric confluence and the portal vein. The mobilization of the pancreas was performed at the superior border of the pancreas. Consequently, the lymph nodes along the common hepatic artery and gastroduodenal artery were dissected. After creating a tunnel behind the neck of the pancreas, the pancreas neck was transected with an endoscopic linear stapler (Endocutter 60 staple, white or blue cartridge; Ethicon Endo-Surgery, Inc, Cincinnati, OH, United States). Dissection of the lymph nodes around the celiac trunk was then performed. Then the splenic artery and splenic vein were divided. The lymph nodes anterior to the aorta between the celiac artery and superior mesenteric artery and those anterior and to the left of the superior mesenteric artery were dissected. The distal pancreas was dissected with soft tissue of retroperitoneum in a medial-to-lateral fashion (Figs. 1, 2). Either the anterior or posterior RAMPS procedure was based on the principles emphasized by Strasberg et al. [1]. After completely resecting the distal pancreas and spleen with en bloc lymph node dissection, the specimen was bagged and retrieved through enlarged umbilical incision. One drainage tubes was left close to the proximal pancreatic remnant. Drainage tubes were routinely removed on postoperative day 3, when amylase of drain fluid was less than 3 times the upper normal serum value. In patients with any measurable volume of drain fluid of amylase-rich (> 3 times the upper normal serum value), drainage tubes were kept in place and removed individually, depending on the enzyme levels.

## Results

We performed 10 consecutive cases of totally LRAMPS. There were six male and four female patients, with a median age of 64 years (range 55–80 years). The mean BMI was 24.1 ± 2.9 kg/m^2^. Two patients underwent previous laparotomy: one underwent cholecystectomy, one appendectomy. Every patient received CT and MRI scanning. A 125 × 108 mm cystic neoplasm in the body of the pancreas with clear margin, multilocular cavity and enhanced internal septum and thick wall was found in one case. The solid mass in the body or tail of the pancreas without invading retroperitoneal space was found the other nine patients. All the pancreatic tumors were apart from celiac axis. EUS with FNA was performed in two cases. Positron emission computed tomography was performed in six cases. Six tumors (60.0%) were located in the pancreatic body and 4 (40.0%) were located in the tail of the pancreas.

No robotic or hand assistance was used. All the patients underwent posterior RAMPS. No patient required conversion and transfusion. The median operative times was 235 min (range 212–270 min), with an EBL of 120 ml (range 100–200 ml). Postoperative complications occurred in 5 (50.0%) patients. Three patients developed a grade B pancreatic fistula requiring persistent drainage longer than 3 weeks. One patient experienced gastric empty delay that was managed conservatively and ultimately cured. One patient developed retroperitoneal infection; he underwent percutaneous drainage. There was no postoperative 30-day mortality and reoperation. The median postoperative hospital stay was 14 days (range 9–24 days).

Nine patients were diagnosed with pancreatic ductal adenocarcinoma, one with pancreatic mucinous cystoadenocarcinoma. Median tumor size was 3.5 cm (range 2–12.5 cm). The median count of retrieved lymph nodes was 15 (range 13–21), and four patients (40%) had malignant-positive lymph nodes. All of these cases achieved a negative tangential margin and R0 resection.

Median follow-up times were 11 months (range 3–14 m). All the patients received the chemotherapy. Two patients developed disease recurrence (pancreatic bed recurrence and liver metastasis) 9 months, 10 months after surgery, respectively. Others survived without tumor recurrence or metastasis.

## Discussion

Laparoscopic distal pancreatectomy (LDP) has been recognized as a standard technique for benign or borderline malignant neoplasms. The findings that LDP is associated with lower estimated blood loss, faster recovery than open distal pancreatectomy have increased interest in the procedure [17, 18]. Due to recent technological developments, LDP has been expanded to treat pancreatic cancer by LRAMPS. But the safety and feasibility of LRAMS for pancreatic cancer remains controversial. This study clarified that LRAMPS is technically safe and feasible procedure in well-selected patients with pancreatic cancer in the distal pancreas.

The morbidity rates of LRAMPS reported in literatures varied greatly from 13.3 to 66.7% (Table 1) [5–11]. PF was the most frequent complication after LRAMPS. The PF rates of LRAMPS varied greatly from 0 to 66.7% (Table 1) [5–11]. Lee et al. [6] reported that laparoscopic or robotic RAMPS had comparable rate of morbidity (25% vs. 37.2%, p = 0.412) and PF (grades B and C; 19.2 vs. 35.7%, p = 0.251) in relation to conventional open distal pancreatosplenectomy. Compared to conventional open distal pancreatosplenectomy, laparoscopic or robotic RAMPS is associated with faster recovery, shorter length of hospital stay (12.3 ± 6.8 vs. 22.4 ± 21.6 days, p = 0.002) [6]. The morbidity rates and PF rates of laparoscopic conventional radical distal pancreatectomy for pancreatic cancer reported in literatures varied greatly from 13.6 to 52.9% [19].Our series with 10 cases showed a morbidity rate of 50.0%, and the PF rate of 30.0%, similar to what have been reported in the literature [5–11]. Even the operation more complicated, the LRAMPS didn't increase the risk of complications but with the advantages related to minimal-access surgery, such as less intraoperative blood loss, faster recovery.

**Table 1:** Main published series of Laparoscopic radical antegrade modular pancreatosplenectomy

Author (year)	N	Operative time (min)	EBL (ml)	Conversion	Morbidity	Pancreatic fistula	Reoperation	Mortality	Postoperative hospital stay (days)	Tumor size (cm)	Count of retrieved lymph nodes	Margin status, RO (%)	Follow-up (months)	Recurrence
Sunagawa et al. (2014) [5]	3	431.0^A^	175^A^	0	1 (33.3%)	1 (33.3%)	0	0	17.3^A^	NA	43^A^	NA	NA	NA
Lee et al. (2014) [6]	12	324.3^A^	445.8^A^	0	3 (25%)	2 (16.6%)	0	0	12.3^A^	2.8^A^	10.5^A^	100	39^B^	5 (41.7%)
Kim et al. (2017) [7]	15	219^A^	250^A^	0	2 (13.3%)	0	0	0	6.1^A^	3.8^A^	18.1^B^	100	46^B^	4 (26.7%)
Ome et al. (2017) [8]	3	358^B^	Minimal to 1 ml	0	1 (33.3%)	1 (33.3%)	0	0	14^B^	NA	NA	NA	NA	NA
Yamamoto et al. (2017) [9]	3	421^B^	75^B^	0	1 (33.3%)	1 (33.3%)	0	0	15^B^	NA	37^B^	100	NA	NA
Xu et al. (2018) [10]	12	250^B^	150^B^	0	8 (66.7%)	8 (66.7%)	1 (8.3%)	0	9^B^	3.4^B^	16^B^	100	10^B^	2 (16.7%)
Kim et al. (2019) [11]	1	220	200	0	0	0	0	0	7	2	21	100	NA	NA
This study	10	235^B^	120^B^	0	5 (50.0%)	3 (30.0%)	0	0	14^B^	3.5^B^	15^B^	100	11^B^	2 (20.0%)

*EBL* estimated blood loss; A = mean; B = median; NA = not available

RAMPS was designed to increase the rate of R0 resection and lymph node yield for pancreatic cancer in the body or tail [1, 2]. Chun et al. [20] performed a systematic literature review that mean lymph node counts of RAMPS was as high as 24, and negative margin rates between 81 and 100%. Tangential margins are reportedly negative in 94% of patients undergoing RAMPS [20]. Studies comparing RAMPS with standard distal pancreatosplenectomy demonstrate significantly higher lymph node counts [21–23]. The lymph node counts of LRAMPS reported in literatures varied greatly from 10.5 to 43 (Table 1) [5–11]. The mean count of retrieved lymph nodes was 18.1 ± 9.5, and 18 patients had malignant-positive lymph nodes [5–11]. Lee et al. [6] reported that laparoscopic or robotic RAMPS had comparable number of retrieved lymph nodes (10.5 ± 7.1 vs. 13.8 ± 11.1, p = 0.313) and R0 resection (100% vs. 85.9%, p = 0.381) in relation to conventional open distal pancreatosplenectomy. The lymph nodes harvested and negative surgical margin of laparoscopic conventional radical distal pancreatectomy for pancreatic cancer reported in literatures varied greatly from 9 to 25.9 and 64.1% to 95.5%, respectively [19]. Our series with 10 cases showed a count of retrieved lymph nodes of 15 (range 13–21) and the R0 resection rate of 100.0%, similar to what have been reported in the literature [5–11]. So whether LRAMPS could achieve better oncological outcomes than laparoscopic conventional distal pancreatosplenectomy or similar oncological outcomes as open RAMPS need more randomized controlled test to confirm.

No study to date has shown improved overall survival between RAMPS and standard distal pancreatectomy [21–23]. Abe et al. [23] reported that median overall survival rates were not significantly different between patients undergoing RAMPS versus standard distal pancreatectomy (47 months vs. 34 months; p = 0.19). In a study of Park et al. [22], on univariate analysis, conventional resection was associated with a worse 5-year overall survival of 12%, compared with 40% after RAMPS (p = 0.014). However, on multivariate analysis, adjuvant chemoradiation and negative margins were the sole factors associated with improved overall survival [22]. Lee et al. [6] reported that there were no significant differences in median overall survival between laparoscopic or robotic RAMPS and conventional open distal pancreatosplenectomy within the Yonsei criteria (60.00 vs. 60.72 months, p = 0.616). So whether the patients could benefit the better survival outcomes after LRAMPS need to be further validated based on additional large-volume studies.

There are several approaches in LRAMPS. Sunagawa et al. [5] and Ome et al. [8] performed a LRAMPS by starting from the resecting the ligament of Treitz and entered the anterior space of the aorta and inferior vena cava. They confirmed that it could be easily to proceed from neck of the pancreas to the level of aorta and easily to avoid causing any damage to the retropancreatic organs, including the left renal vein [5, 8]. Yamamoto et al. [9] developed the artery-first approach LRAMPS for left-sided pancreatic cancer. The artery-first approach means that middle segment of the pancreas was initially separated from both the left renal vein and the superior mesenteric artery with the advantage of early detection of no tumor infiltration into the superior mesenteric artery and the early determination of posterior dissection plane [9]. But in most centers, the dissection plane proceeded vertically during LRAMPS, thereby exposing the left side of the celiac artery and superior mesenteric artery down to the level of the aorta after the division of the neck of the pancreas [6, 7, 10]. We also performed LRAMPS in this manner. In our experience, preoperative accuracy assessment of tumor by CT and MRI and fine operation were the key points of the this manner of LRAMPS.

Whether LRAMPS is the ideal approach for the left-sided pancreatic cancer? Only one retrospective control study of LRAMPS compared with conventional open surgery was indexed on Pubmed [6]. No literature of LRAMPS compared with laparoscopic standard distal pancreatectomy was published. Therefore, a randomized controlled test should be performed to test whether the LRAMPS procedure is superior to open RAMPS or standard distal pancreatectomy. But it was difficult to accomplish owing to the infrequent procedure of LRAMPS [3].

## Conclusions

LRAMPS is technically safe and feasible procedure in well-selected patients with pancreatic cancer in the distal pancreas. The oncologically outcomes need to be further validated based on additional large-volume studies.

## Data Availability

All data generated or analysed during this study are included in this published article.

## Abbreviations

CT: Computed tomography
EBL: Estimated blood loss
EUS: Endoscopic ultrasound
FNA: Fine-needle aspiration
LDP: Laparoscopic distal pancreatectomy
LRAMPS: Laparoscopic radical antegrade modular pancreatosplenectomy
MRI: Magnetic resonance imaging
PF: Pancreatic fistula
RAMPS: Radical antegrade modular pancreatosplenectomy
